# Identification of a Novel EF-Loop in the N-terminus of TRPM2 Channel Involved in Calcium Sensitivity

**DOI:** 10.3389/fphar.2018.00581

**Published:** 2018-06-04

**Authors:** Yuhuan Luo, Xiafei Yu, Cheng Ma, Jianhong Luo, Wei Yang

**Affiliations:** ^1^Department of Neurobiology, Institute of Neuroscience, NHC and CAMS Key Laboratory of Medical Neurobiology, Zhejiang University School of Medicine, Hangzhou, China; ^2^Co-facility Center, Zhejiang University School of Medicine, Hangzhou, China

**Keywords:** TRPM2, calcium, EF-loop, CaM, alanine screen, channel activation

## Abstract

As an oxidative stress sensor, transient receptor potential melastatin 2 (TRPM2) channel is involved in many physiological and pathological processes including warmth sensing, ischemia injury, inflammatory diseases and diabetes. Intracellular calcium is critical for TRPM2 channel activation and the IQ-like motif in the N-terminus has been shown to be important by mediating calmodulin binding. Sequence analysis predicted two potential EF-loops in the N-terminus of TRPM2. Site-directed mutagenesis combining with functional assay showed that substitution with alanine of several residues, most of which are conserved in the typical EF-loop, including D267, D278, D288, and E298 dramatically reduced TRPM2 channel currents. By further changing the charges or side chain length of these conserved residues, our results indicate that the negative charge of D267 and the side chain length of D278 are critical for calcium-induced TRPM2 channel activation. G272I mutation also dramatically reduced the channel currents, suggesting that this site is critical for calcium-induced TRPM2 channel activation. Furthermore, D267A mutant dramatically reduced the currents induced by calcium alone compared with that by ADPR, indicating that D267 residue in D267–D278 motif is the most important site for calcium sensitivity of TRPM2. In addition, inside-out recordings showed that mutations at D267, G272, D278, and E298 had no effect on single-channel conductance. Taken together, our data indicate that D267–D278 motif in the N-terminus as a novel EF-loop is critical for calcium-induced TRPM2 channel activation.

## Introduction

As a member of the mammalian TRP channel family, transient receptor potential melastatin 2 (TRPM2) channel is a calcium-permeable, non-selective cation channel, which is assembled by four subunits surrounding a central ion-permeating pore and each subunit has six transmembrane segments with its N- and C-termini in the cytoplasmic face of the cell (Perraud et al., 2001). This channel is widely expressed in a variety of cells, including neurons (Ye et al., 2014; Ostapchenko et al., 2015; Demirdaş et al., 2017), microglia (Haraguchi et al., 2012; Syed Mortadza et al., 2015; Huang et al., 2017), pancreatic β-cells (Yosida et al., 2014; Ito et al., 2017), monocytes and macrophages (Wehrhahn et al., 2010; Zou et al., 2013), endothelial cells (Hecquet et al., 2008; Mittal et al., 2017) and pericytes (Jiang et al., 2017). Accumulative evidence supports that TRPM2 is a cellular oxidative stress sensor (Di et al., 2011; Yu et al., 2015; Wang et al., 2016) and it has important functions in various physiological and pathophysiological processes. For example, TRPM2 plays an important role in bacterial clearance partially due to its ability to activate cytokine generation (Knowles et al., 2011; Qian et al., 2014). In addition, recent studies have reported TRPM2 as a temperature sensor mediating the warmth sensing (Song et al., 2016; Tan and Mcnaughton, 2016). More importantly, many studies show that TRPM2 channel mediates ROS-induced cell death through permeating excessive calcium into cells, which results in many kinds of diseases including ischemia injury, diabetes and inflammatory diseases (Jiang et al., 2010; Syed Mortadza et al., 2015).

Although it is still debatable whether NAD metabolites are ligands at the TRPM2 channel (Lange et al., 2008; Tóth et al., 2015), there is consensus that TRPM2 can be activated by intracellular adenosine diphosphate-ribose (ADPR) (Perraud et al., 2005; Moreau et al., 2013), calcium (Du et al., 2009; László and Beáta, 2009) and H_2_O_2_ (Hecquet et al., 2008; Bari et al., 2009). Our recent study has provided a better delineation of the ADPR binding pocket in the NUDT9 homology (NUDT9-H) domain of TRPM2 (Yu et al., 2017), and several studies have demonstrated strong synergy of ADPR and calcium (Perraud et al., 2001; Lange et al., 2008; Tóth and Csanády, 2010). More importantly, TRPM2 channel activation by ADPR strongly depends on calcium, particularly intracellular calcium (Starkus et al., 2007), suggesting calcium is crucial for TRPM2 channel activation. Interestingly, calcium is able to open the TRPM2 channel independent of ADPR or the NUDT9-H domain (Du et al., 2009). These observations strongly indicate intracellular calcium regulation on TRPM2 channel function is complex. Although accumulating evidence showed the importance of calcium in the TRPM2 channel gating, the molecular mechanism is not fully understood. So far, studies in the literature have postulated mainly two mechanisms through which calcium regulates ion channels or receptors: (1) calcium directly binds to the ion channel, such as the large-conductance voltage-and calcium-activated K^+^ channel (Yusifov et al., 2008; Miranda et al., 2016), calcium-activated chloride channel (Tien et al., 2014), and transient receptor potential polycystin-3 channel (Hu et al., 2015); (2) calcium indirectly opens the channel through binding to calmodulin (CaM), including small-conductance K_Ca_ channel (Xia et al., 1998; Schumacher et al., 2001) and transient receptor potential vanilloid 4 channel (Loukin et al., 2015). Previous studies have identified an IQ-like motif in the N-terminus of the TRPM2 channel that interacts with CaM and is responsible for the sensitivity of channel activation by calcium (Tong et al., 2006; Du et al., 2009). However, there are clues that also pointed to direct regulation of TRPM2 channel by calcium, for example, the TRPM2 channel exhibited fast opening by calcium in inside-out recording under which there is no CaM in the bath solution (Tóth and Csanády, 2010; Tóth et al., 2015). Therefore, it is important to address whether there are calcium-binding sites in the TRPM2 channel to improve our understanding in the channel gating mechanisms.

Previous studies have described several calcium-binding motifs including EF-hand. The EF-hand motif is a helix-loop-helix structure or motif, which contains a Ca^2+^-binding loop (EF-loop) (Pidcock and Moore, 2001; Gifford et al., 2007). Accumulating evidence has shown the EF-loop can be classified into canonical and non-canonical EF-loop (Gifford et al., 2007). The canonical EF-loop consists of 12 residues, among which the first and the last residues with high conservation make great contribution to calcium binding, and the middle site provides a sharp bend that is necessary for calcium binding to the 5th and 7th sites of the loop (Krebs and Heizmann, 2007). The EF-hand has been identified in ion channels, including transient receptor potential ion channel polycystin 2 (Petri and Marks, 2010; Yang et al., 2015), transient receptor potential polycystin-3 channel (Hu et al., 2015), and Ca^2+^ release-activated Ca^2+^ channel (Srikanth et al., 2010). Mutation or deletion of the EF-loop can strongly affect the channel sensitivity to calcium (Hu et al., 2015).

By comparing with the known EF-hand sequences, we find two potential EF-loop motifs in the N-terminus of TRPM2. Furthermore, our sequence alignment suggests that the first EF-loop presents the characteristic of a canonical EF-loop, while the second EF-loop is a non-canonical EF-loop but highly conserved across TRPM2, TRPM4 and TRPM8. Functional assay in combination with alanine screen indicate several conserved residues in two potential EF-loops are also critical for TRPM2 activation by calcium. Further mutation assays show that highly conserved residues D267, D278, and G272 in the first EF-loop are critical for calcium-induced TRPM2 channel activation, suggesting the D267–D278 motif might be a novel EF-loop and uncovering a new mechanism for TRPM2 channel activation by calcium.

## Materials and Methods

### Cell Culture and Molecular Biology

HEK-293T cells were cultured in DMEM medium (Gibco, United States) supplemented with 10% FBS (Gibco, United States), 100 units/mL penicillin, and 100 mg/mL streptomycin at 37°C in a humidity-controlled incubator with 5% CO_2_. The cDNA encoding the human TRPM2 (hTRPM2) was kindly provided by Dr. A. M. Scharenberg (University of Washington, Seattle, WA, United States). Mutations were introduced by site-directed mutagenesis and confirmed by full coding sequence (CDS) sequencing. Cells were transiently transfected with wildtype (WT) hTRPM2 or its mutants using Lipofectamine 2000 based on the manufacturer’s recommended transfection protocols. The cDNA of GFP was co-transfected as a marker for the transfected cells. HEK-293T cells were used for electrophysiological experiments 24 h after transfection. Chemicals and reagents used were purchased from Sigma unless otherwise indicated.

### Electrophysiology

All patch-clamp recordings were performed at room temperature, using an EPC10 amplifier (HEKA) which was controlled by PatchMaster software. Data were acquired at 20 kHz and filtered offline at 50 Hz. Series resistance (Rs) was compensated up to 90% to reduce series resistance. Cells in which Rs was >20 MΩ were discarded.

For whole-cell recordings, voltage ramps with 500 ms duration from -100 mV to 100 mV were applied every 5 s. The currents at -80 mV were denoted by circles in figures, and the current values at +80 mV were used to calculate current density. The current–voltage (I–V) curves were obtained from the current responses to voltage ramps. Patch electrodes were pulled from borosilicate glass (Sutter Instrument) and had resistances of 3–5 MΩ when filled with internal solutions. The saturated ADPR solution (500 μM) of internal pipette: 147 mM NaCl, 1 mM MgCl_2_, 0.05 mM EGTA, 10 mM Hepes and 500 μM ADPR (pH 7.4, adjusted with NaOH). The high calcium solution of internal pipette: 50 mM CaCl_2_, 75 mM NaCl, 1 mM MgCl_2_, 10 mM Hepes (pH 7.4, adjusted with NaOH). The intracellular solution containing 10 or 30 μM ADPR plus 1 μM free Ca^2+^: 145 mM Cs-methanesulfonate (CsSO_3_CH_3_), 8 mM NaCl, 1 mM EGTA, 10 mM Hepes, 0.7794 mM CaCl_2_ (pH 7.2, adjusted with CsOH). Ca^2+^ was calculated by Maxchelator. The standard extracellular solution contained 147 mM NaCl, 2 mM KCl, 1 mM MgCl_2_, 2 mM CaCl_2_, 10 mM Hepes, and 13 mM glucose (pH 7.4, adjusted with NaOH). At the end of each recording, 20 μM N-(*p*-amylcinnamoyl) anthranilic acid (ACA) (Sigma) was applied. The cells whose currents completely inhibited by ACA were used in analysis.

Single-channel recordings were carried out under inside-out configuration, using glass pipettes of 6–7 MΩ and at a holding potential of -80 mV. The pipette solution contained 145 mM NaCl, 5.6 mM KCl, 2 mM MgCl_2_, 1.2 mM CaCl_2_, 10 mM Hepes (pH 7.2, adjusted with NaOH), and the bath solution contained 140 mM K-gluconate, 4 mM NaCl, 2 mM MgCl_2_, 10 mM Hepes, 1 mM EGTA, 0.7794 mM CaCl_2_, and 30 μM ADPR (pH 7.2, adjusted with KOH). For all experiments, Change in extracellular solutions was performed by RSC-200 (Biologic) with a solution change time of ∼100 ms. Single channel events were displayed as all point histograms, and single channel conductance were estimated based on the resolvable unitary currents as illustrated in **Figure 13**.

### Biotinylation Assay and Western Blot

Biotinylation assays and Western blot were conducted as previously described (Yu et al., 2017). In brief, after transfection with WT hTRPM2 and mutants for 24–36 h, HEK293T cells were rinsed with ice-cold PBS. The cells were subsequently incubated with a fresh preparation of 1 mg/ml Sulfo-NHS-SS-Biotin (Thermo Fisher Scientific) dissolved in PBS with Tween-20 at 4°C for 30 min. Subsequently, unreacted biotin was quenched with PBS containing 100 mM glycine on ice. The cells were lysed with RIPA buffer (10 mM Tris, 150 mM NaCl, 1 mM EDTA, 0.1% SDS, 1% Triton X-100, and 1% sodium deoxycholate, pH 7.4) and subjected to centrifugation at 12,000 *g* for 15 min. The resulting supernatant was incubated with 40 μl of 50% slurry of NeutrAvidin beads (Thermo Fisher Scientific) for 2 h at 4°C with continuous rotation. After several washes with RIPA buffer, the biotinylated proteins were eluted from the NeutrAvidin beads with 60 μl of 2 × SDS sample buffer. The primary antibody used was rabbit anti–TRPM2 (1:1000; 96785; Abcam), anti-actin (1:5000; G-9545; Sigma), and the secondary antibody was goat anti–rabbit IgG-HRP (1:10,000; 31420; Thermo Fisher Scientific). Enhanced Chemiluminescence (ECL; Pierce) was used for detection of signal, and images were captured by using a Fuji LAS-3000 Imaging System.

### Data Analysis and Statistics

Data were analyzed with patchmaster (HEKA) and IgorPro (Wave-Metrics). All results, where appropriate, are presented as mean ± SEM. Curve fitting was performed using Origin software and singe channel conductance analysis using IgorPro (Wave-Metrics). Statistical comparisons were made by using one-way ANOVA with Bonferroni correction. *P* < 0.05 indicated statistical significance.

## Results

### Activation of TRPM2 by Calcium in Synergy With ADPR

**FIGURE 1:**
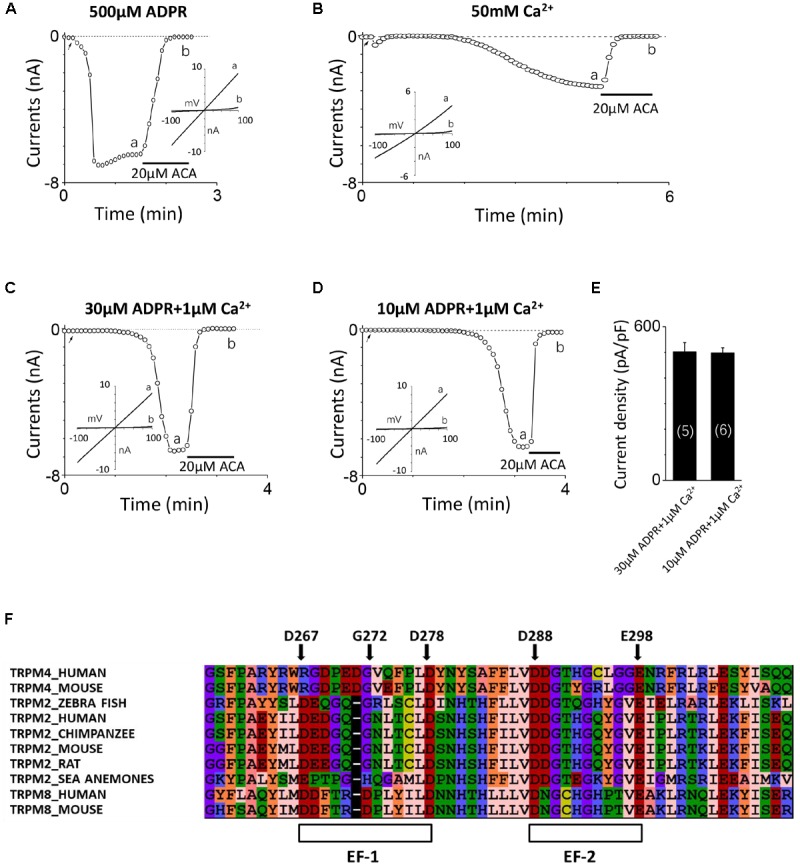
TRPM2 activation by intracellular Ca^2+^ and ADPR, and identification of potential EF-loops in TRPM2. **(A–D)** The representative TRPM2 channel current recordings elicited by **(A)** 500 μM ADPR, **(B)** 50 mM Ca^2+^**, (C)** 30 μM ADPR plus 1 μM Ca^2+^, and **(D)** 10 μM ADPR plus 1 μM Ca^2+^ from cells expressing the hTRPM2 plasmid. The black lines represent application of 20 μM ACA. The arrow in each panel indicates the time point at which whole cell configuration was established. The insets in **(A–D)** show I/V curves at time points indicated by a and b. **(E)** Summary of the current density induced by 30 μM ADPR plus 1 μM Ca^2+^ and 10 μM ADPR plus 1 μM Ca^2+^ in HEK293T cells expressed human TRPM2 channels. The numbers of cells examined in each case are indicated in parentheses. **(F)** Sequence alignments of the N-terminus including human TRPM2 (O94759), zebra fish TRPM2 (S5TZ89), chimpanzee TRPM2 (H2QL45), mouse TRPM2 (Q91YD4), rat TRPM2 (E9PTA2), sea anemones TRPM2 (jgi.Nemve1.248535| estExt_fgenesh1_pg.C_6220005), human TRPM4 (Q8TD43), mouse TRPM4 (Q7TN37), human TRPM8 (Q7Z2W7) and mouse TRPM8 (Q8R4D5).

Consistent with our previous study (Yang et al., 2010), the TRPM2 channel currents were induced by 500 μM ADPR applied in the intracellular solution and inhibited by 20 μM ACA (**Figure 1A**). Since previous study reported calcium can activate the TRPM2 channel alone (Du et al., 2009), we initially added 100 μM Ca^2+^ instead of ADPR into the intracellular solution attempting to induce TRPM2 channel currents, but no current was detected (data not shown). After gradually increasing the calcium concentration, the TRPM2 currents were successfully induced by 50 mM Ca^2+^, which exhibited the typical linear I–V curve of the TRPM2 channel and sensitivity to inhibition by ACA (**Figure 1B**). Although these data confirmed intracellular calcium could activate the TRPM2 channel alone, the concentration of calcium used was too high to be suitable for investigating the physiological or pathological relationships between TRPM2 and calcium.

It is well known that ADPR induced the TRPM2 channel activation through interacting with the NUDT9-H domain in the C-terminus of TRPM2 (Perraud et al., 2001; Kühn and Lückhoff, 2004), and previous studies have demonstrated that calcium has a synergy effect with ADPR to open the TRPM2 channel (Lange et al., 2008; Du et al., 2009). The TRPM2 channel currents induced by 30 or 10 μM ADPR in the absence of calcium were very small (Yu et al., 2017) but were increased dramatically when 1 μM Ca^2+^ was added (**Figures 1C,D**), suggesting that calcium indeed facilitated ADPR-induced TRPM2 channel activation. As **Figure 1E** showed, the mean current of TRPM2 induced in these conditions is similar. The internal pipette solution contained 30 μM ADPR and 1 μM Ca^2+^ was used in alanine mutagenesis screen (which induced TRPM2 currents more quickly, **Figure 1C**), and the internal pipette solution contained 10 μM ADPR and 1 μM Ca^2+^ was used in functional assay of multiple mutations at single site (in order to enlarge the contribution of calcium, **Figure 1D**).

### There Are Two Potential EF-Loops in the N-terminus of TRPM2

To determine whether the TRPM2 channel contains the potential calcium-binding sites, we aligned the sequences of TRPM2, TRPM4 and TRPM8 and found two potential EF-loops in the N-terminus of the channel (**Figure 1F**). For the first one, D267-D278 motif contains 12 residues, and eight of them are in accordance with the sequence of a canonical EF-loop. Moreover, the first (D267), sixth (G272), and last (D278) residues in this motif are consistent with the canonical EF-loop where they are highly conserved (more than 90%). We also noticed four residues in this loop which did not exist in previous reported EF-loops that showed an obvious lower conservation (no more than 52%) (Gifford et al., 2007). As for the second one, D288–E298 motif consists of 11 residues as a non-canonical EF-loop. Although it has the negatively charged residues at the first (D288) and the last (E298) residues, it shows a lower degree of conservation compared with D267–D278 motif as an EF-loop. Therefore, we were interested in determining whether these potential EF-loops were involved in the TRPM2 channel activation by calcium.

### Alanine Screening Showed Several Residues in Two Potential EF-Loops Are Important for Calcium-Induced TRPM2 Channel Activation

To explore the involvement of D267–D278 and D288–E298 motifs in TRPM2 channel activation induced by calcium, we performed alanine screening for all residues in these motifs by measuring their TRPM2 channel currents using whole-cell recording in HEK-293T cells expressing individual mutants.

**FIGURE 2:**
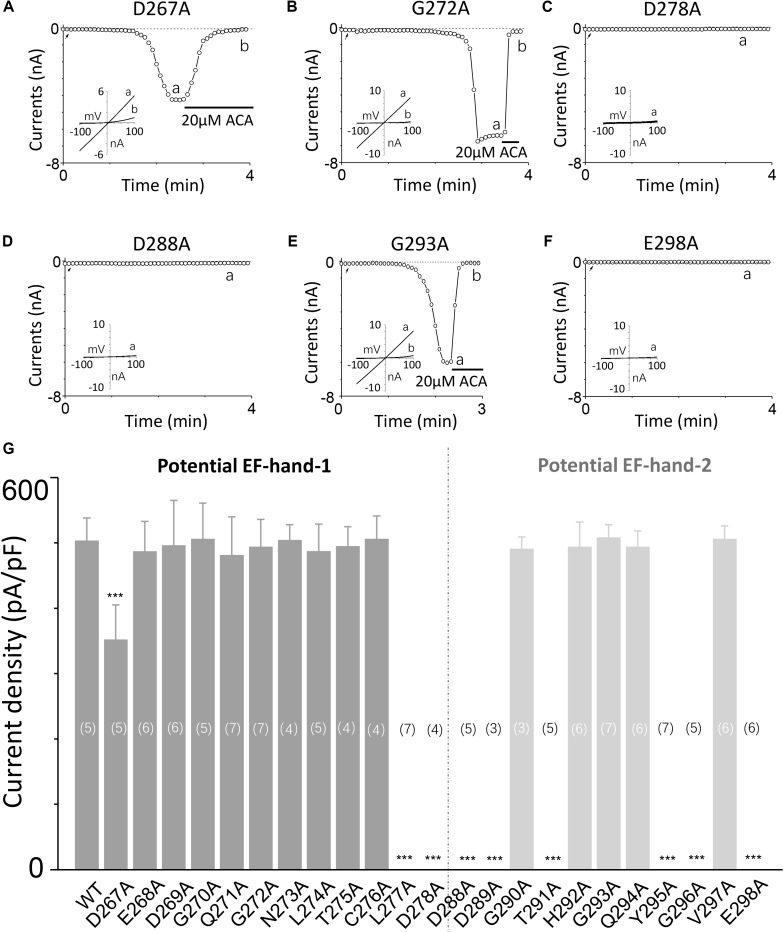
Alanine mutagenesis screen of D267–D278 and D288–E298 motifs determines their effects on the TRPM2 channel activation by calcium. **(A–F)** Representative whole-cell recordings of TRPM2 channel currents induced by 30 μM ADPR plus 1 μM Ca^2+^ from HEK293T cells expressing human **(A)** D267A, **(B)** G272A, **(C)** D278A, **(D)** D288A, **(E)** G293A, **(F)** E298A TRPM2 channels. The arrow in each panel indicates the time point at which whole cell configuration was established. The insets in **(A–F)** show the I/V curves at time points indicated by a and b. **(G)** Summary of the current density induced by 30 μM ADPR plus 1 μM Ca^2+^ in cells expressing WT and mutant TRPM2 channels. The dotted line separates the two potential EF-loops. The numbers of cells examined in each case are indicated in parentheses. ^∗∗∗^*p* < 0.001 compared with WT.

**FIGURE 3:**
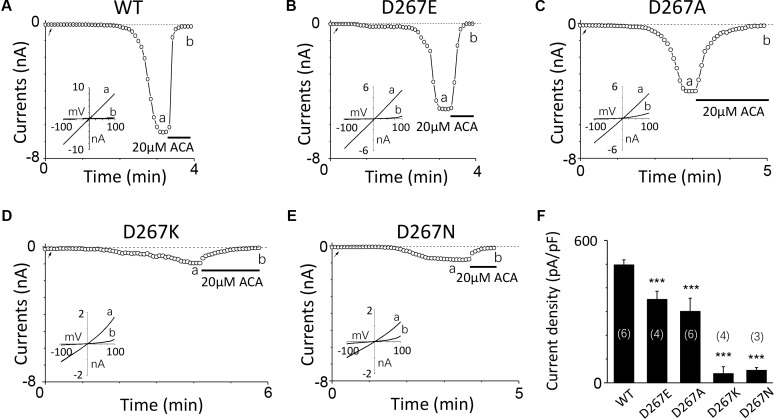
Multiple substitutions at D267 indicate that its charge is involved in calcium sensitivity. **(A–E)** Representative whole-cell recordings of TRPM2 channel current induced by 10 μM ADPR plus 1 μM Ca^2+^ from HEK293T cells expressing human **(A)** WT, **(B)** D267E, **(C)** D267A, **(D)** D267K, **(E)** D267N TRPM2 channels. The arrow in each panel indicates the time point at which whole cell configuration was established. The insets in **(A–E)** show I/V curves at time points indicated by a and b. **(F)** Summary of the current density in **(A–E)**. The numbers of cells examined in each case are indicated in parentheses. ^∗∗∗^*p* < 0.001 compared with WT.

Our results showed alanine substitution of D267 significantly reduced the channel current (**Figures 2A**, **3C**), whereas the G272A mutant had normal function (**Figure 2B**). However, the D278A mutant completely failed to respond to calcium and ADPR (**Figure 2C**), suggesting that the first EF-loop may be involved in calcium sensing. In the second predicted EF-loop, there were no channel currents at both D288A and E298A (**Figures 2D,F**). Similarly, although Gly at the middle site of the second predicted EF-loop has a high conservation of 96% (Gifford et al., 2007), alanine substitution of G293 had no significant influence on the channel current (**Figure 2E**). As expected, the I-V curves of all the functional mutant channels remained the same as the WT channel (**Figures 2A–F**). In addition, mutations of several other residues located at the second potential EF-loop including D289, T291, Y295, and G296 rendered complete loss of channel function (**Figure 2G**). As most of them were less conserved, the second predicted EF-loop is less likely to be a Ca^2+^ sensor. Our data taken together provide evidence suggesting that both sequences are involved in the TRPM2 channel gating.

### The First Potential EF-Loop Plays a Major Role in Calcium-Induced TRPM2 Channel Activation

In the canonical EF-loop, residues with high conservation participate in calcium binding mainly by several ways: negative charge, hydrogen bond, Van der Waals force, or helping to maintain the structure (Krebs and Heizmann, 2007). In order to confirm whether these two potential EF-loops affect the TRPM2 channel activation through calcium and find the way of these conserved sites interacting with calcium, we further made various mutations which altered the charge or size of their side chains.

Firstly, we mutated Asp267 to Glu in the D267-D278 motif, which slightly increased the side chain length without changing negative charge property, and the current of this mutant was reduced (**Figures 3A,B**). Conversely, Asn mutation at D267, which neutralized the charge without effect on its side chain length, reduced the TRPM2 channel current significantly (**Figure 3E**), suggesting the negative charge at D267 influences calcium sensitivity. Furthermore, charge-reversing mutation in D267K, showed similar effect with D267N (**Figures 3D,F**), confirming that the charge of this residue is critical for calcium-induced TRPM2 channel activation.

**FIGURE 4:**
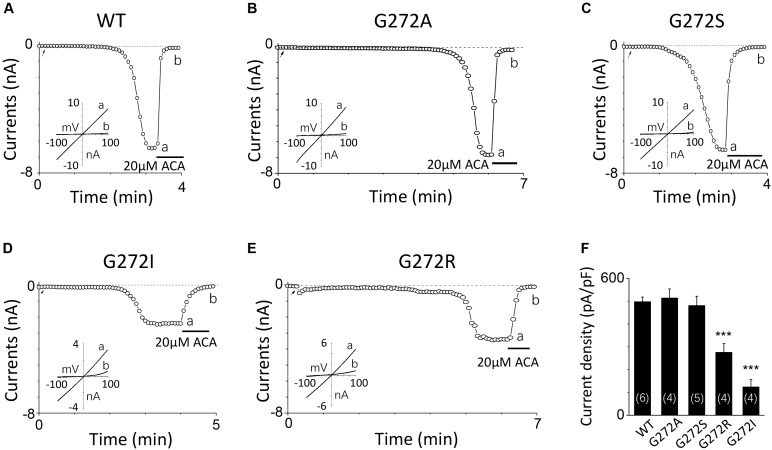
Mutations of G272 have minor effects on the TRPM2 channel activation by calcium. **(A–E)** Representative whole-cell recordings of TRPM2 channel currents induced by 10 μM ADPR plus 1 μM Ca^2+^ from HEK293T cells expressing human **(A)** WT, **(B)** G272A, **(C)** G272S, **(D)** G272I, **(E)** G272R TRPM2 channels. The arrow in each panel indicates the time point at which whole cell configuration was established. The insets in **(A–E)** show I/V curves at time points indicated by a and b. **(F)** Summary of the current density in **(A–E)**. The numbers of cells examined in each case are indicated in parentheses. ^∗∗∗^*p* < 0.001 compared with WT.

For G272, a highly conserved residue in the middle of the D267-D278 motif, increase in its side chain length didn’t affect the channel activation. For instance, G272A and G272S mutant channels behaved the same as the WT channel (**Figures 4A–C**). However, substitution with residues possessing large side chain, such as G272R and G272I, dramatically decreased the currents to half or even less compared with that of WT (**Figures 4D–F**), indicating G272 may also participate in the TRPM2 channel activation by calcium, but the effect of this site may be different from that of D267.

**FIGURE 5:**
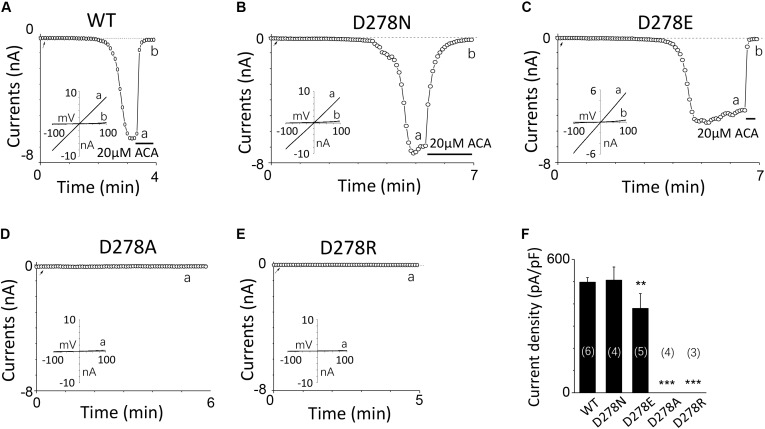
Multiple substitutions at D278 indicate that its side chain length is critical for calcium sensitivity. **(A–E)** Representative whole-cell recordings of TRPM2 channel currents induced by 10 μM ADPR plus 1 μM Ca^2+^ from HEK293T cells expressing human **(A)** WT, **(B)** D278N, **(C)** D278E, **(D)** D278A, **(E)** D278R TRPM2 channels. The arrow in each panel indicates the time point at which whole cell configuration was established. The insets in **(A–E)** show I/V curves at time points indicated by a and b. **(F)** Summary of the current density in **(A–E)**. The numbers of cells examined in each case are indicated in parentheses. ^∗∗^*p* < 0.01 and ^∗∗∗^*p* < 0.001 compared with WT.

**FIGURE 6:**
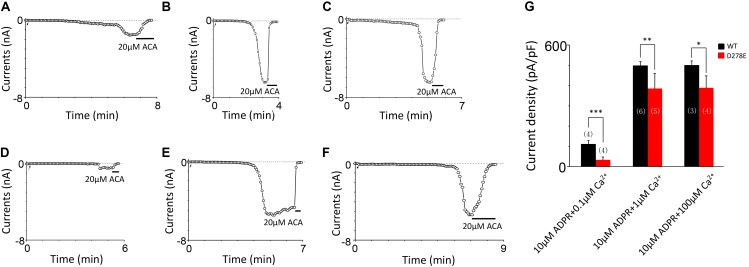
D278E impaired the calcium sensitivity of TRPM2 in Ca^2+^ dose dependence. **(A–C)** Representative whole-cell recordings of TRPM2 channel currents induced by **(A)** 10 μM ADPR plus 0.1 μM Ca^2+^, **(B)** 10 μM ADPR plus 1 μM Ca^2+^ **(C)** 10 μM ADPR plus 100 μM Ca^2+^ from HEK293T cells expressing human WT TRPM2 channels. **(D–F)** Representative whole-cell recordings of TRPM2 channel currents induced by **(D)** 10 μM ADPR plus 0.1 μM Ca^2+^, **(E)** 10 μM ADPR plus 1 μM Ca^2+^ **(F)** 10 μM ADPR plus 100 μM Ca^2+^ from HEK293T cells expressing human D278E mutant. The arrow in each panel indicates the time point at which whole cell configuration was established. **(G)** Summary of the current density in **(A–F)**. The numbers of cells examined are indicated in parentheses. ^∗^*p* < 0.05, ^∗∗^*p* < 0.01, and ^∗∗∗^*p* < 0.001 compared with WT.

For D278, removal of its negative charge with mutation D278N made the mutant channel remain the same as WT (**Figures 5A,B**). In contrast, D278E, which changed the size of the side chain with the negative charge preserved, produced a significant reduction in the channel currents (**Figure 5C**). These data suggest that the side chain length at D278 play a major role in determining calcium-induced channel activation. The most substantial changes were induced by the D278A and D278R, both of them altered the length of the side chain and removed or reversed its charge at the same time, which resulted in switching the channel to be non-functional (**Figures 5D–F**), suggesting that in addition to the side chain length, the charge at this residue also affects the channel activation. Furthermore, our data showed the current of D278E mutant induced by 10 μM ADPR plus 100 μM Ca^2+^ is similar to that induced by 10 μM ADPR plus 1 μM Ca^2+^ (**Figure 6**), suggesting that this dose is saturated concentration. However, 10μM ADPR plus 0.1μM Ca^2+^ induced a much smaller TRPM2 current, indicating D278E mutant is activated by Ca^2+^ in a dose dependent manner (**Figure 6**). More importantly, the current of D278E is much smaller than that of WT TRPM2 induced by 10 μM ADPR plus 0.1 μM Ca^2+^, which indicated D278E in the first EF-loop affected the Ca^2+^ induced TRPM2 activation.

**FIGURE 7:**
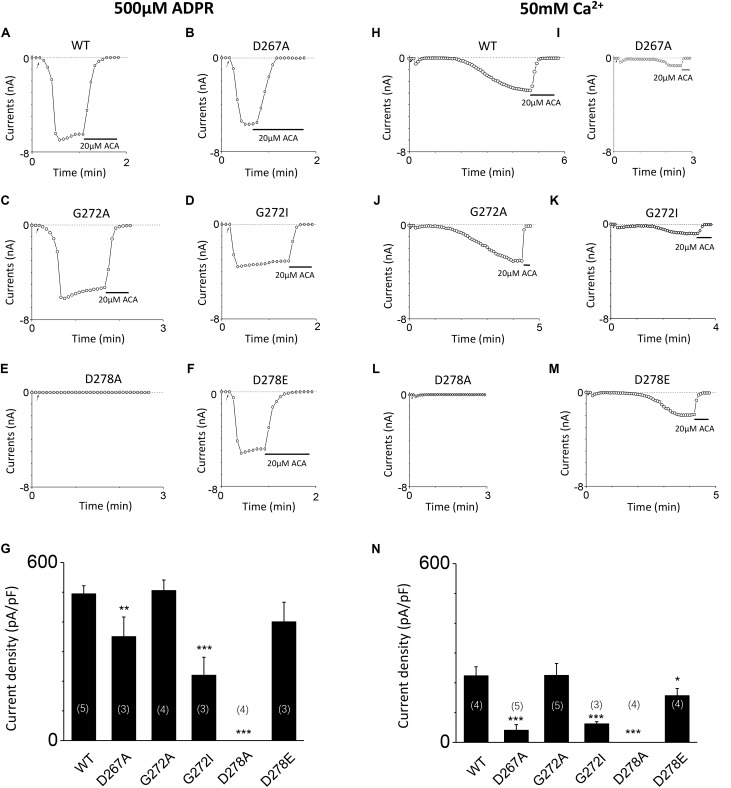
Mutants altered the calcium sensitivity of TRPM2 activated by either ADPR or calcium alone. **(A–F)** Representative whole-cell recordings of TRPM2 channel current induced by 500 μM ADPR from HEK293T cells expressing human **(A)** WT, **(B)** D267A, **(C)** G272A, **(D)** G72I, **(E)** D278A, **(F)** D278E TRPM2 channels. The arrow in each panel indicates the time point at which whole cell configuration was established. **(G)** Summary of the current density in **(A–F). (H–M)** Representative whole-cell recordings of TRPM2 channel current induced by 50 mM Ca^2+^ from HEK293T cells expressing human **(H)** WT, **(I)** D267A, **(J)** G272A, **(K)** G272I, **(L)** D278A, **(M)** D278E TRPM2 channels. The arrow in each panel indicates the time point at which whole cell configuration was established. **(N)** Summary of the current density in **(H–M)**. The numbers of cells examined in each case are indicated in parentheses. ^∗^*p* < 0.05, ^∗∗^*p* < 0.01, and ^∗∗∗^*p* < 0.001 compared with WT.

To further clarify the effect of these mutants on calcium sensitivity of TRPM2, we examined the response of D267A, G272A, G272I, D278A, and D278E to saturated ADPR (500 μM). Our data indicates the currents of these mutants are similar to that of these mutants induced by 10 μM ADPR plus 1 μM Ca^2+^ treatment (**Figures 7A–G**), supporting that TRPM2 activated by ADPR needs intracellular calcium (Tóth and Csanády, 2010). To directly determine whether these mutants mediated the calcium sensitivity, we detected the response of these mutants to 50 mM calcium (**Figures 7H–N**). Our results showed the current density of D267A, G272I, and D278E mutants were much less than that of WT TRPM2 induced by 50 mM Ca^2+^, supporting that the conserved residues in the first EF-loop is critical for calcium sensitivity of TRPM2. Similar to previous studies (Du et al., 2009; Yu et al., 2017), the current density of WT TRPM2 induced by 50 mM Ca^2+^ alone is much smaller than that induced by 500 μM ADPR (**Figures 7G,N**). We also observed the current density of the D267A and G272I mutants were much less than that of WT TRPM2 induced by 500 μM ADPR, so to clearly examine the specific contribution of these mutants on the calcium sensitivity of TRPM2, we normalized the mean current densities induced by 50 mM Ca^2+^ to that by 500 μM ADPR. Interestingly, we found that the ratio for D267A (0.116) was much smaller than such ratios for WT (0.453) and other mutants (0.445 for G272A, 0.284 for G272I, and 0.392 for D278E). We observed that the ratio of G272A mutant is similar to that of WT TRPM2, suggesting this site is less important for calcium sensitivity. However, the ratio of D267A mutant largely decreased compared with WT TRPM2, indicating the D267 site is most important for calcium sensitivity of TRPM2.

**FIGURE 8:**
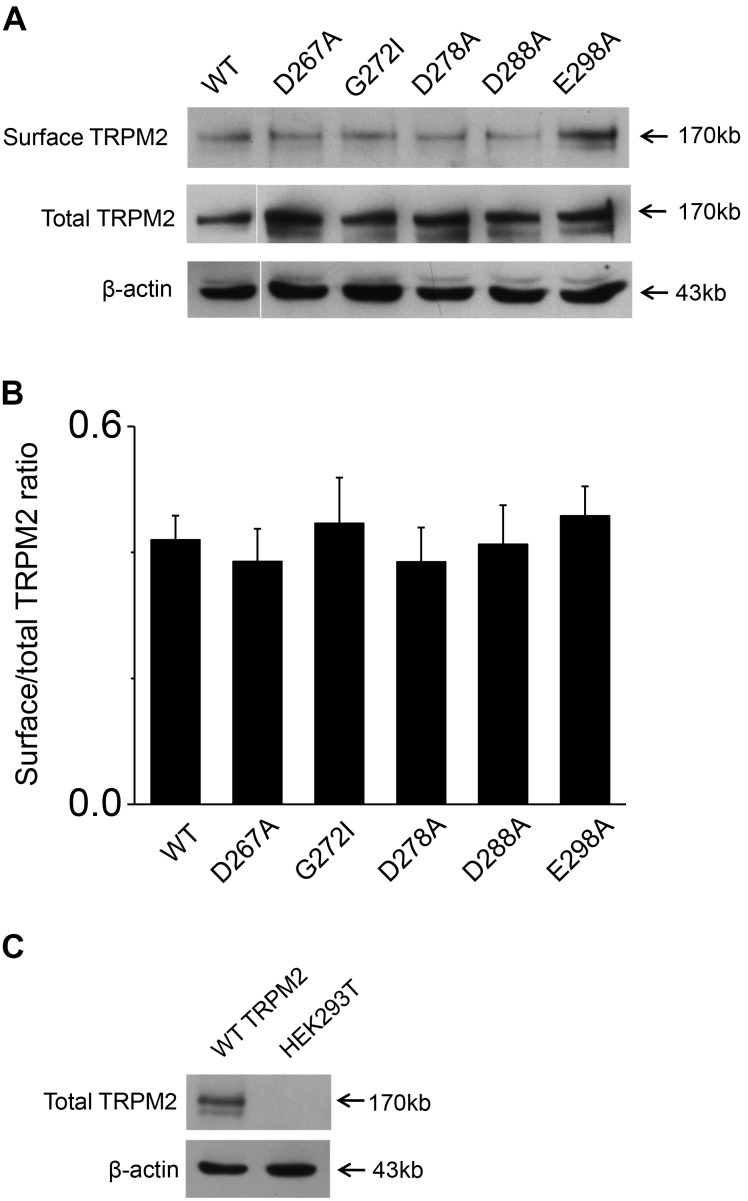
Biotinylation assay for surface expression of the TRPM2 and its mutants. **(A)** The five mutants including D267A, G272I, D278A, D288A, and E298A that impaired channel function was detected the surface expression in HEK293T cells. The top panel shows surface expression, the middle panel shows total expression, and the bottom panel shows actin expression. Arrows indicate the band of TRPM2, the indicated TRPM2 mutants and actin. **(B)** Statistical analysis of Westernblotting data was calculated as the relative ratio of surface TRPM2 or its mutants normalized to total TRPM2 or its mutants (*n* = 3), no significant difference between TRPM2 and its mutants. **(C)** Rabbit anti–TRPM2 (1:1000; 96785; Abcam) specifically recognizes TRPM2. The top panel shows total TRPM2 expression, and the bottom panel shows actin expression. Arrows indicate the band of TRPM2 and actin.

**FIGURE 9:**
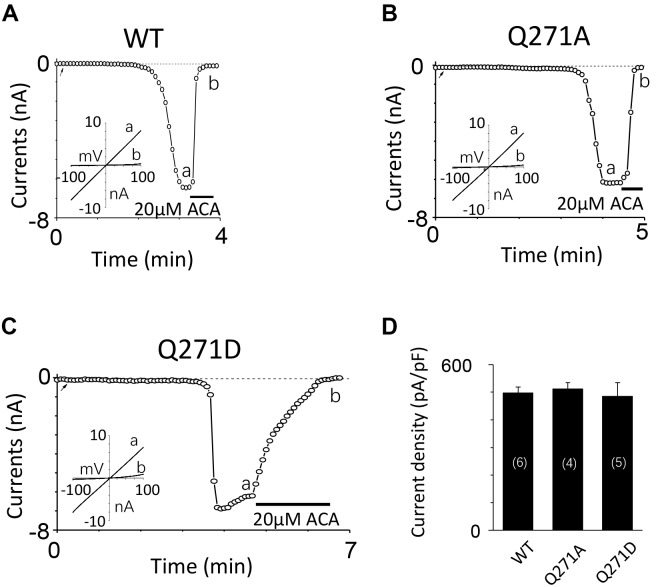
Mutations at Q271 had no effect on the TRPM2 channel activation. **(A–C)** Representative whole-cell recordings of TRPM2 channel currents induced by 10 μM ADPR plus 1 μM Ca^2+^ from HEK293T cells expressing human **(A)** WT, **(B)** Q271A, **(C)** Q271D TRPM2 channels. The arrow in each panel indicates the time point at which whole cell configuration was established. The insets in **(A–C)** show I/V curves at time points indicated by a and b. **(D)** Summary of the current density in **(A–C)**. The numbers of cells examined in each case are indicated in parentheses.

To exclude the possibility of these mutants affected surface expression, we performed the biotinylation assay for several critical mutants (**Figures 8A,B** and Supplementary Figures 1, 2), our data showed these mutants did not change the surface expression pattern. Our control experiment also showed the TRPM2 antibody specifically recognized TRPM2 (**Figure 8C** and Supplementary Figures 3, 4). Except the conserved residues studied above, we also examined other less conserved residues. For example, Q271A and Q271D had no effect on the TRPM2 channel currents (**Figure 9**).

Taken together, our data strongly support the first EF-Loop is critical for calcium sensitivity of TRPM2.

### The Second Potential EF-Loop May Be Critical for TRPM2 Gating Process

**FIGURE 10:**
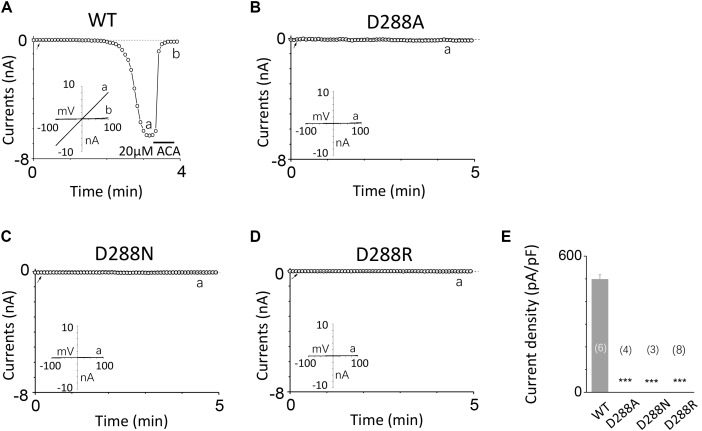
Mutations at D288 resulted in non-function. **(A–D)** Representative whole-cell recordings of TRPM2 channel currents induced by 10 μM ADPR plus 1 μM Ca^2+^ from HEK293T cells expressing human **(A)** WT, **(B)** D288A, **(C)** D288N, **(D)** D288R TRPM2 channels. The arrow in each panel indicates the time point at which whole cell configuration was established. The insets in **(A–D)** represent I/V curves at time points indicated by a and b. **(E)** Summary of the current density in **(A–D)**. The numbers of cells examined in each case are indicated in parentheses. ^∗∗∗^*p* < 0.001, when compared with WT.

**FIGURE 11:**
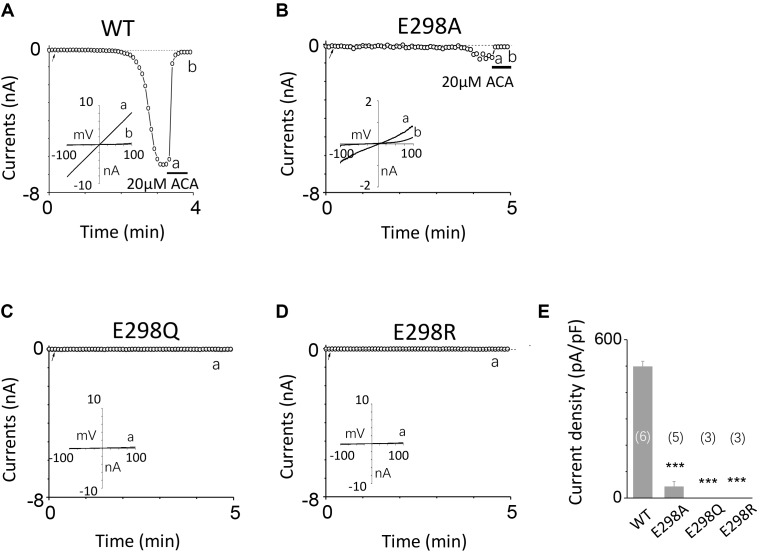
Effects of E298 mutations on channel activation. **(A–D)** Representative whole-cell recordings of TRPM2 channel currents induced by 10 μM ADPR plus 1 μM Ca^2+^ from HEK293T cells expressing human **(A)** WT, **(B)** E298A, **(C)** E298Q, **(D)** E298R TRPM2 channels. The arrow in each panel indicates the time point at which whole cell configuration was established. The insets in **(A–D)** represent I/V curves at time points indicated by a and b. **(E)** Summary of the current density in **(A–D)**. The numbers of cells examined in each case are indicated in parentheses. ^∗∗∗^*p* < 0.001, when compared with WT.

**FIGURE 12:**
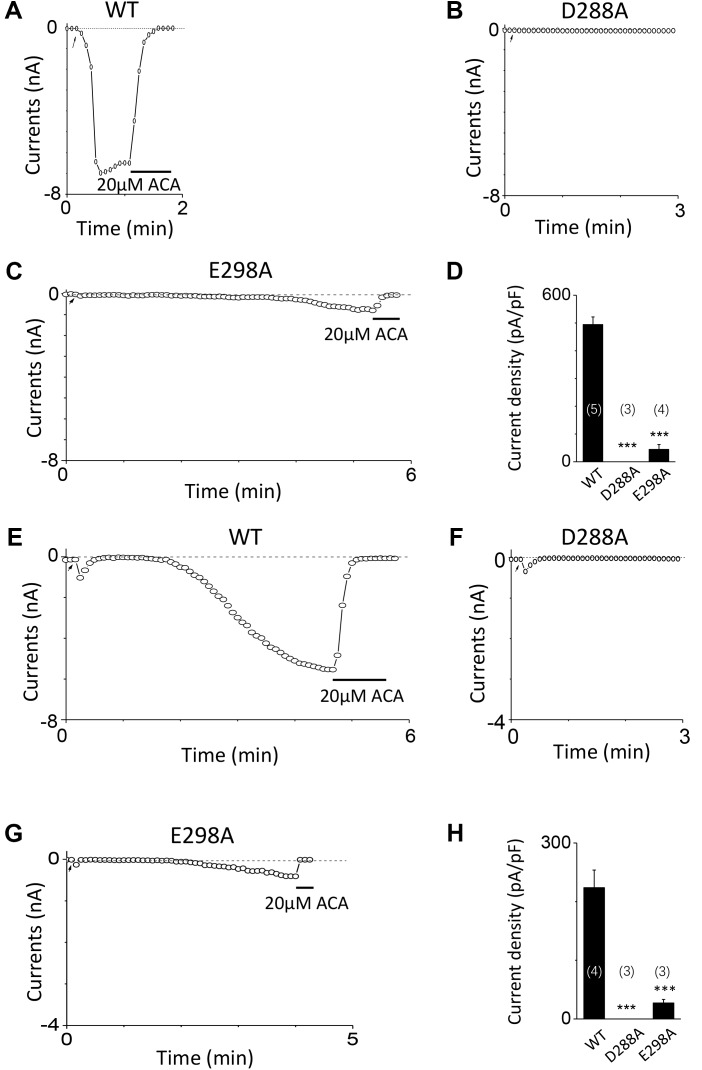
Response of D288A and E298A to saturated ADPR or calcium. **(A–C)** Representative whole-cell recordings of TRPM2 channel currents induced by 500 μM ADPR from HEK293T cells expressing human **(A)** WT, **(B)** D288A, **(C)** E298A. **(D)** Summary of the current density in **(A–C)**. **(E–G)** Representative whole-cell recordings of TRPM2 channel currents induced by 50 mM Ca^2+^ from HEK293T cells expressing human **(E)** WT, **(F)** D288A, **(G)** E298A. **(H)** Summary of the current density in **(E–G)**. The arrow in each panel indicates the time point at which whole cell configuration was established. The numbers of cells examined in each case are indicated in parentheses. ^∗∗∗^*p* < 0.001, when compared with WT.

To investigate the function of the second potential EF-loop in calcium-induced TRPM2 channel activation, we chose to examine D288 and E298, the two most conserved residues in the non-canonical EF-loop (**Figure 1F**) by introducing various mutations. Unexpectedly, all of the mutations at D288 (D288A, D288N, D288R) failed to be activated by calcium (**Figure 10**), which hindered us to further explain the role of this site in TRPM2 activation. As for E298, while E298A mutation dramatically reduced the TRPM2 channel currents (**Figures 11A,B**), charge-neutralizing mutations in E298Q and charge-reversing mutation in E298R failed to respond to calcium and ADPR (**Figures 11C–E**). Furthermore, the current of both D288A and E298A induced by 500 μM ADPR or 50 mM Ca^2+^ is similar to that induced by 10 μM ADPR plus 1 μM Ca^2+^ (**Figure 12**), which hinder us to determine whether the second potential EF-loop is involved in the calcium sensitivity of TRPM2.

### Mutations of Conserved Residues Do Not Change Single Channel Conductance

**FIGURE 13:**
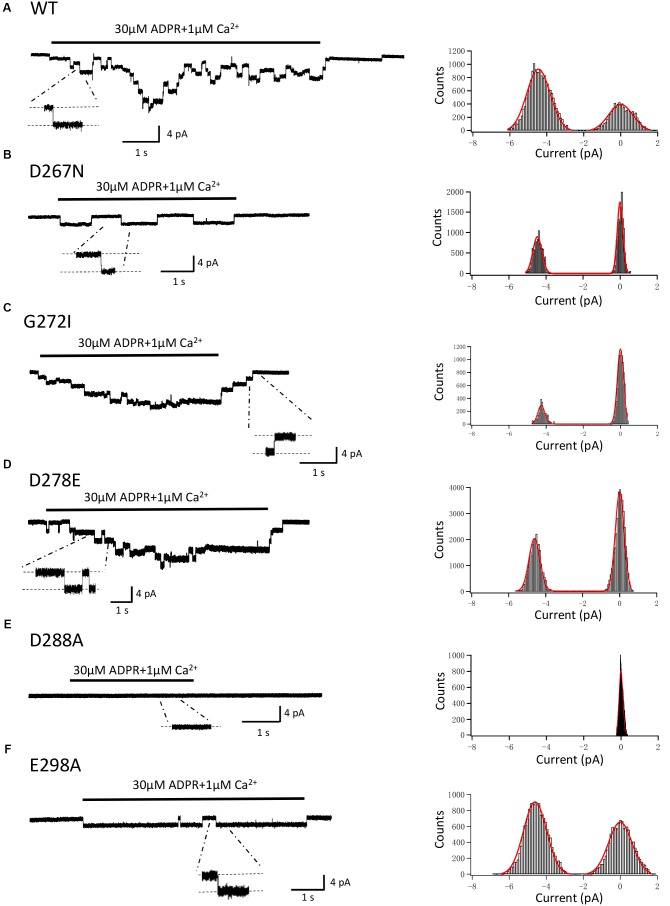
Single-channel events of TRPM2 and its mutants. Right panel, representative single-channel currents elicited by application of 30 μM ADPR plus 1 μM Ca^2+^ to the cytosolic side of inside-out patches excised from HEK293T cells expressing human **(A)** WT, **(B)** D267N, **(C)** G272I, **(D)** D278E, **(E)** D288A, **(F)** E298A TRPM2 channels. Single channel events are clearly seen in the expanded traces, to which the scales are applicable. The resolvable unitary currents at –80 mV were ∼4 pA. Left panel, all-point histograms of the current amplitudes from indicated recordings as illustrated in the right.

In order to exclude the possibility that mutations may alter the single channel conductance, as well as to confirm the previous studies which showed direct interaction between calcium and the TRPM2 channel (László and Beáta, 2009; Tóth and Csanády, 2010; Tóth et al., 2015), we performed the inside-out recordings in HEK-293T cells expressing WT or mutant hTRPM2 channels. The resolvable unitary channel currents at -80 mV were about 4 pA for the WT channel induced by 30 μM ADPR and 1 μM Ca^2+^ (**Figure 13A**), which were consistent with our previous study (Yang et al., 2010). While D288A failed to show any single channel currents, D267N, G272I, D278E, and E298A mutants had similar single channel current amplitude as the WT channel (**Figures 13B–F**), indicating mutations of these residues did not affect single channel conductance.

## Discussion

Calcium plays a critical role in the TRPM2 channel gating process, especially in activating the TRPM2 channel alone or in synergy with ADPR (Du et al., 2009). How could calcium regulate the TRPM2 channel activation has not been fully understood. An IQ-like domain in the N-terminus of TRPM2 has been shown to bind with CaM, and thereby activates or facilitates ADPR-induced activation of the TRPM2 channel (Du et al., 2009). Interestingly, several studies have also implied calcium directly interacts with the TRPM2 channel (László and Beáta, 2009; Tóth and Csanády, 2010; Tóth et al., 2015). However, no calcium binding site in TRPM2 has been identified yet. In order to address whether there exists a calcium binding site in TRPM2, we performed the sequence alignment for the species of TRPM2, and found two potential EF-loop motifs in N terminus of TRPM2. Through mutational manipulations of the charge, length of the side chain or both at the highly conserved residues, our results demonstrate that mutations, especially at the first and the last residues in these EF-loop sequences, affect the channel activation by calcium. Taken together, we predict two potential EF-loops in the N-terminus of the TRPM2 channel, our results further show the D267–D278 motif as a canonical EF-loop mediating calcium sensitivity of TRPM2. Within this motif, D267 is the most critical site for calcium sensitivity of TRPM2.

In the canonical EF-loops, mutations at the highly conserved residues can abolish its ability to bind calcium (Morohashi et al., 2002; Celić et al., 2008), which has been used as a method to identify the existence of EF-loop. In the D267–D278 motif, currents became smaller as the charge of the first residue D267 was altered. Among the mutants examined, the mutant with charge-conserving substitution of D267E exhibited the largest currents while the currents for mutants carrying charge-neutralizing substitution of D267N, and charge-reversing substitution of D267K were dramatically decreased. Although the current of D267A is similar to that of D267E in both saturated ADPR and 10 μM ADPR plus 1 μM Ca^2+^ conditions, the response of D267A to high Ca^2+^ is largely reduced. These results indicate that the negative charge of D267 is important for calcium sensitivity of TRPM2. Compared with D267, mutations at G272 induced much milder influence on the channel currents. Both G272A and G272S mutant channels functioned similarly as the WT TRPM2 channel, and the currents of which were decreased but still detectable when it was replaced by the arginine or isoleucine. These data suggest G272 may form the sharp bend of the structure rather than directly interact with calcium, as previously reported (Krebs and Heizmann, 2007). In this case, the flexibility and small size of this residue are more important than its charge for calcium interaction. Unlike D267, D278A but not D278N disrupted the channel function induced by calcium, which suggests that the side chain length at D278 is much more pivotal than its charge. These data taken together with the results of inside-out recordings, suggest that the mutations of these highly conserved residues in the first potential EF-loop change the channel sensitivity to calcium without effect on single channel conductance. Our biotinylation assay showed the surface expression of these mutants is similar to that of TRPM2. Taken together, above results make the D267–D278 motif more likely to be an EF-loop where calcium binding is mainly determined together by the negative charge of D267 and the side chain of D278. In addition, G272 may help to maintain the proper structure of the EF-loop. Since there has been no structure information of the TRPM2 channel, the exact process of how this motif is involved in Ca^2+^ binding still needs to be determined in the future.

Unlike the D267–D278 motif, the second potential EF-loop consists of 11 residues as a non-canonical EF-loop and replacement with alanine of more than half of the residues made the channel fail to be activated any more. Because of the high conservation showed in the sequence alignment, it might be an important motif for the TRPM2 channel activation. Moreover, there was no current as a result of any of the mutations introduced at D288, from both whole-cell and inside-out recordings, but has normal surface expression. Mutations at E298 also significantly disrupted TRPM2 channel activation without any effect on the channel expression. However, these mutants with no or little function hindered us from further exploring their involvement in calcium-induced TRPM2 channel activation. Moreover, these two residues are highly conserved not only in TRPM2, but also in TRPM4 and TRPM8, it is well known that TRPM8 is not activated by intracellular calcium, which reduced the possibility of this motif for TRPM2 activated by calcium.

Our recent study has identified several residues in the C-terminal NUDT9-H domain that are important in mediating the interaction between ADPR and TRPM2, and mutations of such residues do not have influence on the TRPM2 channel gating by calcium (Yu et al., 2017), suggesting that calcium can activate the channel alone. Inside-out recordings in our study together with the research by Tóth and Csanády (2010) and Tóth et al. (2015) demonstrate that in an environment free of CaM, calcium can open the channel directly. Since co-expressing of CaM mutant with mutations at four EF-hands decreased but did not eliminated the TRPM2 channel currents (Du et al., 2009), we propose that calcium may activate TRPM2 in both direct and indirect ways, which provides a much more precise regulation of TRPM2 channel activation. Similar situation also appears in calcium-activated chloride channels, for which some studies demonstrate that calcium activates the channel by itself (Yu et al., 2012; Terashima et al., 2013; Tien et al., 2014), while others support that CaM is responsible for channel activation (Tian et al., 2011; Jung et al., 2013; Vocke et al., 2013). Similarly, accumulating studies suggest that the binding sites for CaM or calcium in TRPM2 channel may be not unique. For example, previous study suggested that there is a calcium binding site in the deep crevices near the pore (László and Beáta, 2009). In our study, the two potential EF-loops are identified by sequence alignment, and our data from functional assay further suggest that the first EF-loop is mainly involved in the TRPM2 channel activation by calcium. Considering several mutations with no current in the D288–E298 motif and its high conservation in different TRPM channels, we propose it more likely to regulate the TRPM2 gating process instead of calcium sensitivity.

In summary, we have identified a conserved EF-loop (D267-D278) in the TRPM2 channel in the N-terminus which contributes to the calcium sensitivity. It will be of great interest in dissecting its contributions in TRPM2-mediated physiological and pathological processes in the future. Our results also expand the understanding of the EF-loop mediated mechanisms of calcium regulation of ion channels.

## Author Contributions

WY, YL, and XY designed and performed the experiments and analyzed the data. CM performed sequence alignments. WY, YL, and JL conceived the study and wrote the manuscript.

## Conflict of Interest Statement

The authors declare that the research was conducted in the absence of any commercial or financial relationships that could be construed as a potential conflict of interest.
