# Supplementary material for: Identification of a Novel EF-Loop in the N-terminus of TRPM2 Channel Involved in Calcium Sensitivity
Source: Front Pharmacol. 2018 Jun 4;9:581. doi: 10.3389/fphar.2018.00581 (PMC5994415; doi:10.3389/fphar.2018.00581)
Supplement: Supplementary file 1 [file Presentation_1.pdf]

Identification of a novel EF-loop in the N-terminus of TRPM2 channel involved in calcium sensitivity

Yuhuan Luo, Xiafei Yu, Cheng Ma, Jianhong Luo, Wei Yang*

Department of Neurobiology, Institute of Neuroscience, NHC and CAMS Key Laboratory of Medical Neurobiology, Zhejiang University School of Medicine, Hangzhou, China

*** Correspondence:**
Wei Yang
yangwei@zju.edu.cn

Supplementary Figures

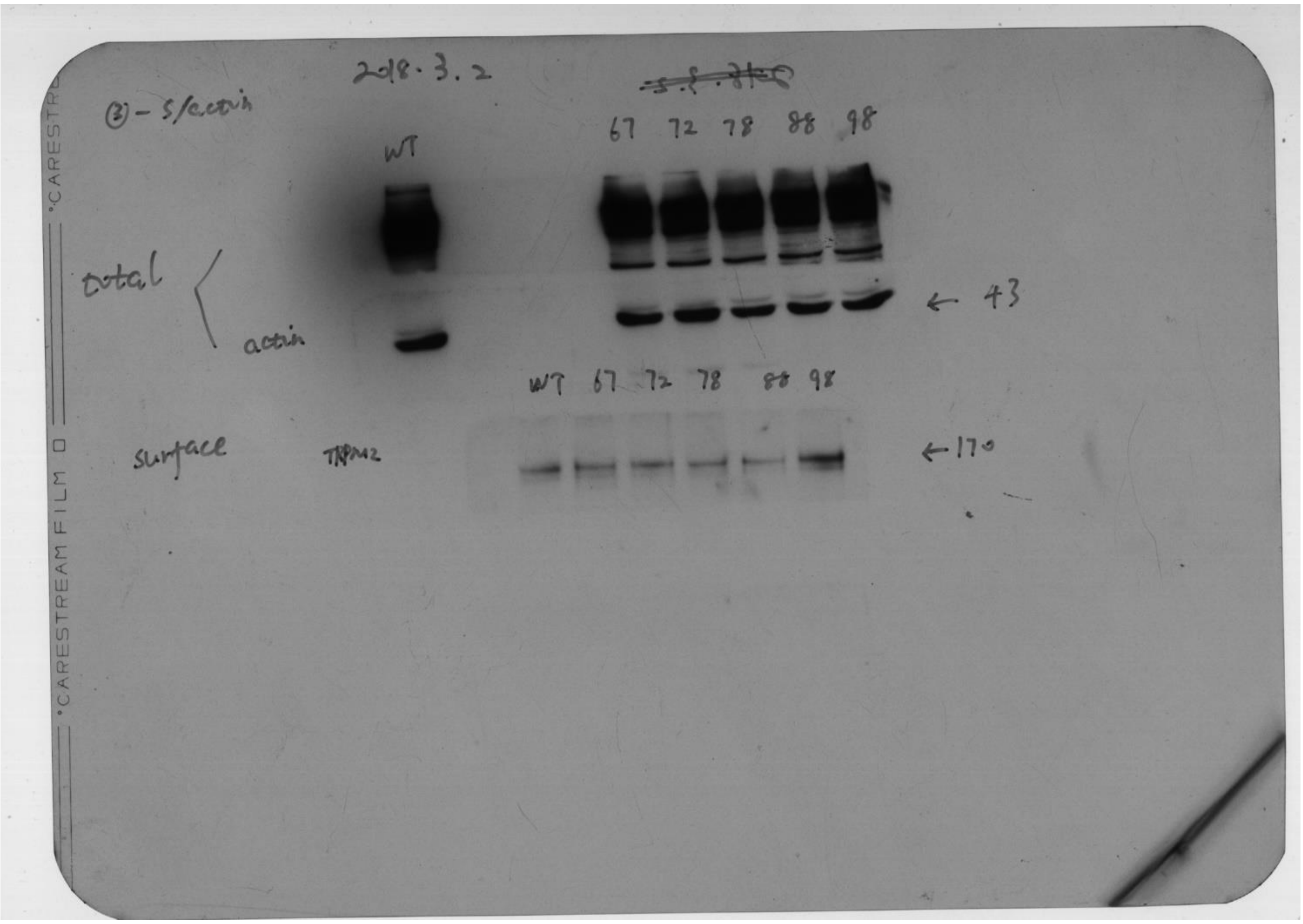

Supplementary Figure 1. A full scan of the entire original gel for Figure 8A (surface TRPM2 and actin)

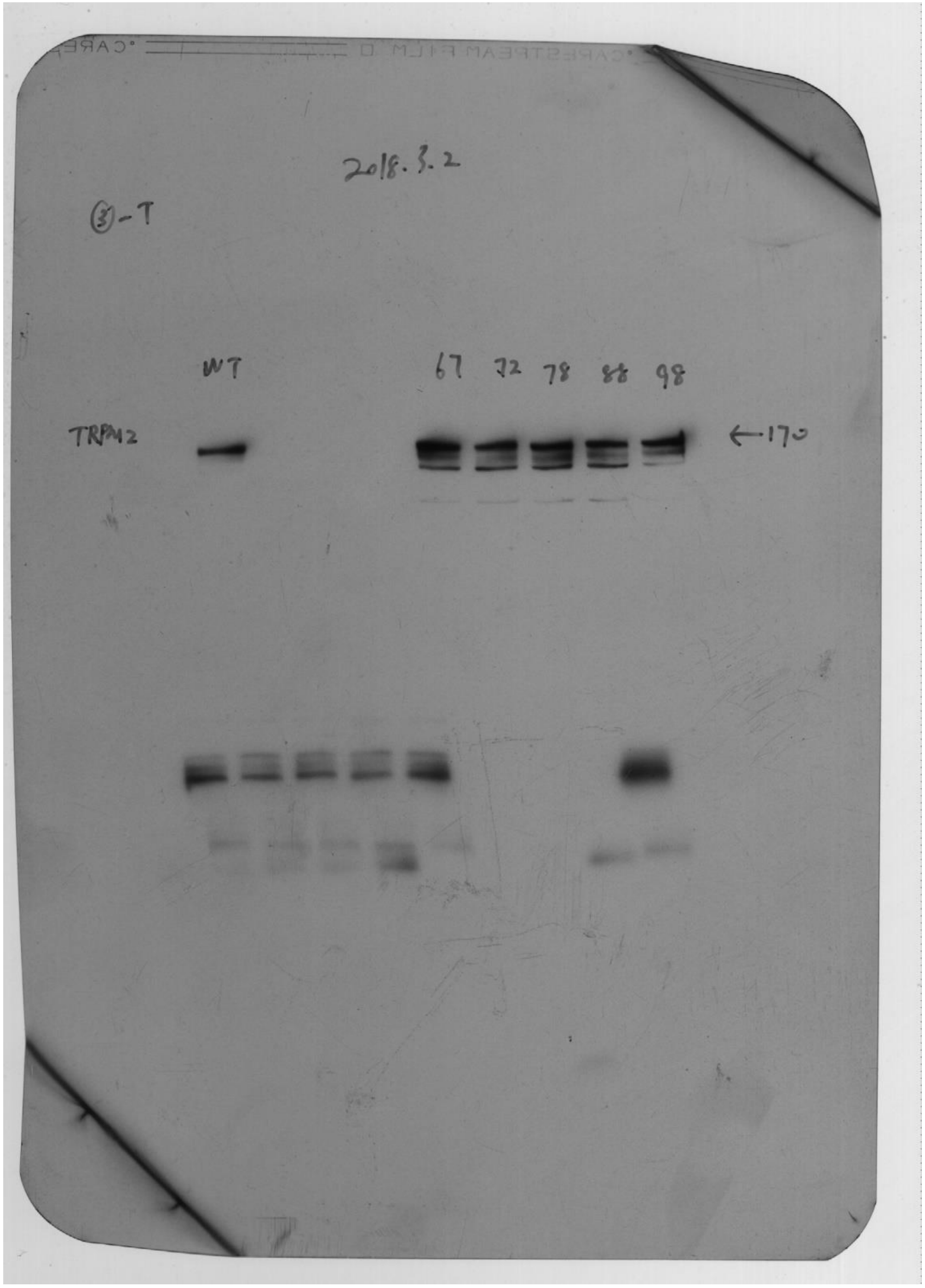
2018.3.2
③-T
WT
67
72
78
88
98
TRPM2
←170

Supplementary Figure 2. A full scan of the entire original gel for Figure 8A (total TRPM2)

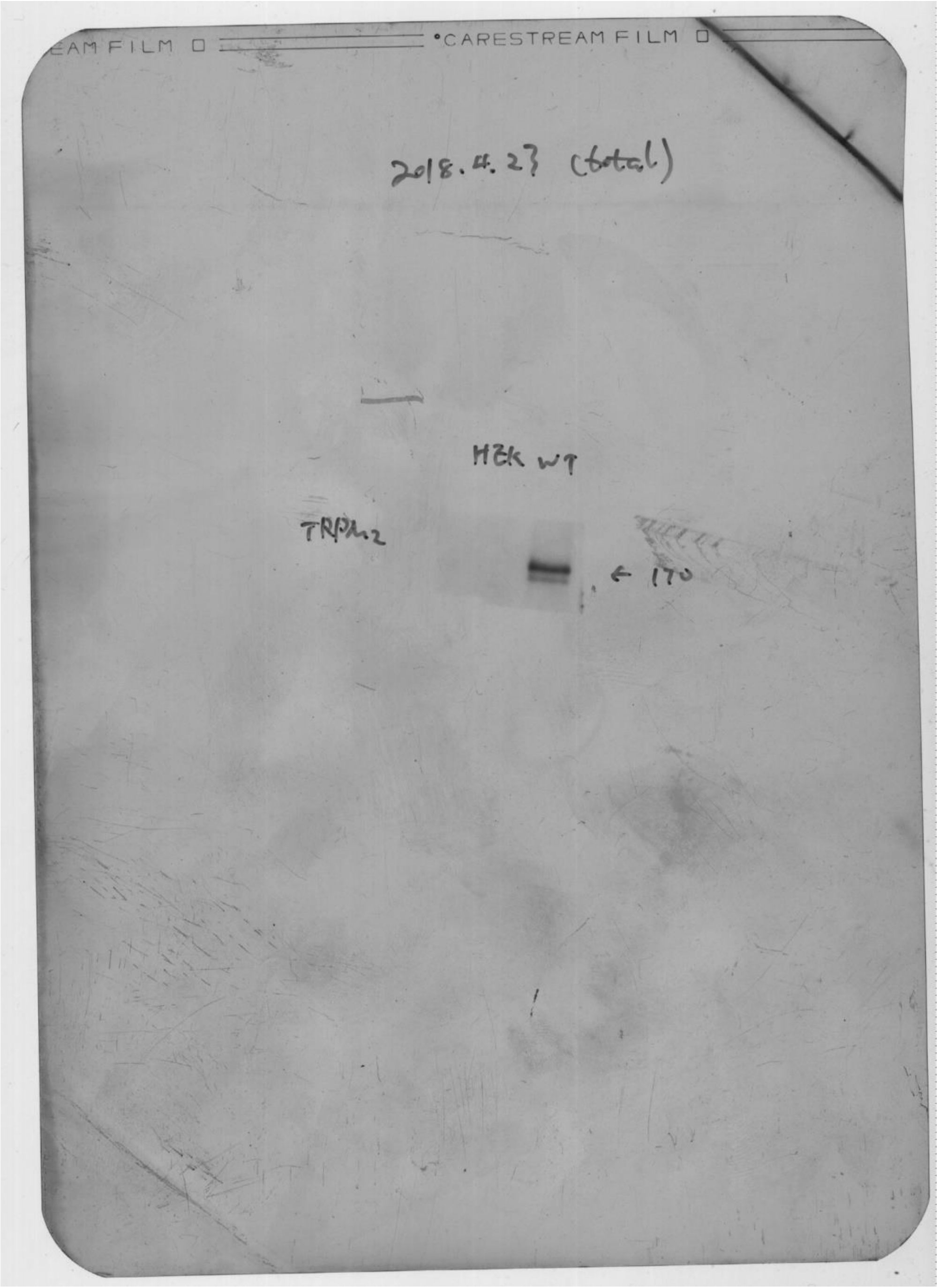

Supplementary Figure 3. A full scan of the entire original gel for Figure 8C (total TRPM2)

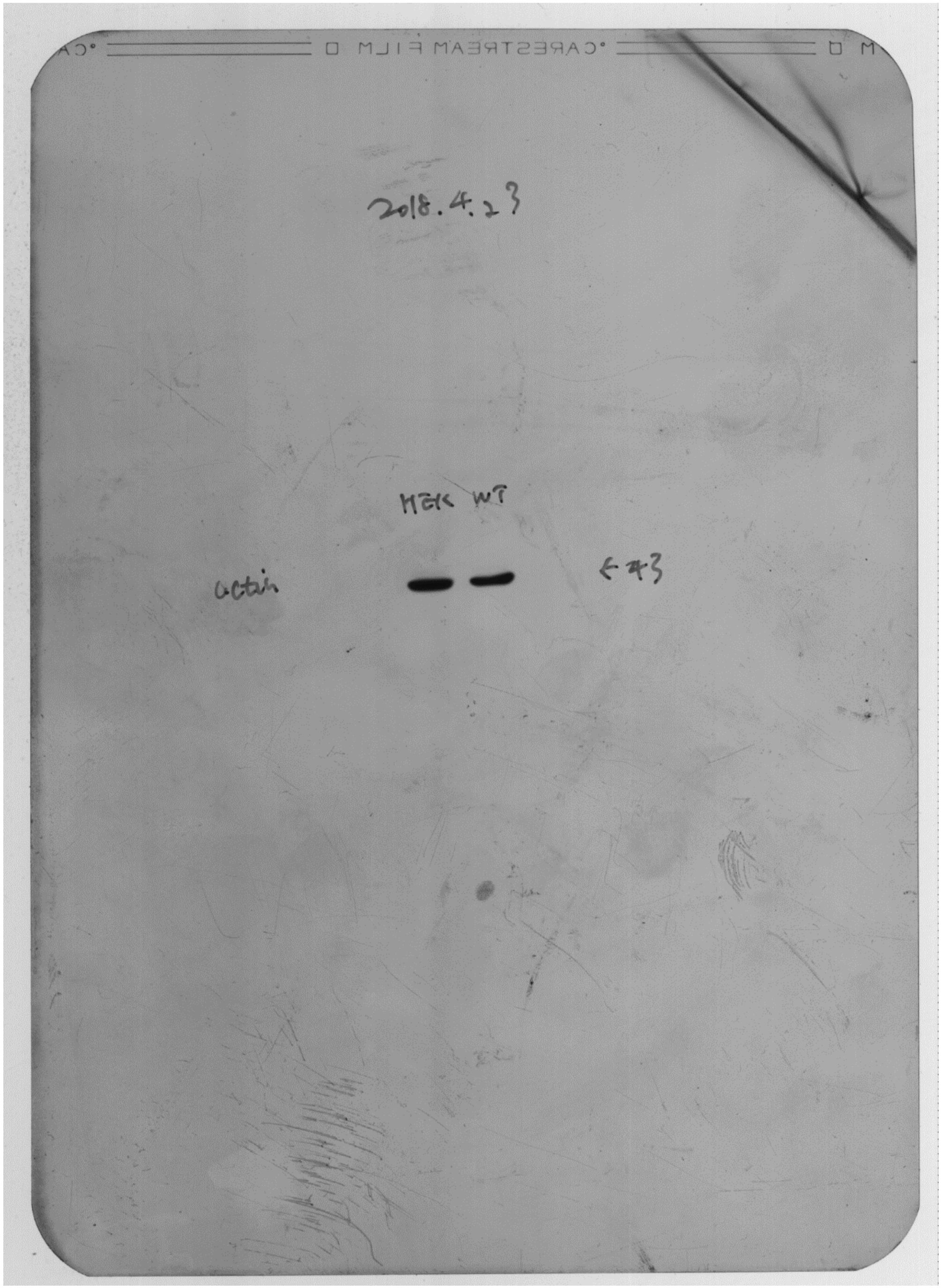

Supplementary Figure 4. A full scan of the entire original gel for Figure 8C (actin)
